# Echography-guided Botulinum Toxin for Moving Ear Syndrome

**DOI:** 10.5334/tohm.951

**Published:** 2024-11-11

**Authors:** Vidal Yahya, Rosa Consuelo Chavez, Laura Campiglio, Manuela Zardoni, Alberto Priori

**Affiliations:** 1 Aldo Ravelli Center for Neurotechnology and Experimental Brain Therapeutics, Department of Health Sciences, University of Milan, Milan, Italy; 2 Clinical Neurology Unit, ASST Santi Paolo e Carlo, San Paolo University Hospital Milan, Italy

**Keywords:** moving ear syndrome, dystonia, tic, phenomenology, botulinum toxin

## Abstract

**Background:**

Moving Ear Syndrome is a rare hyperkinetic disorder.

**Phenomenology shown:**

This Video Abstract illustrates typical backward movements of the right ear associated with pain and discomfort in a man with Moving Ear Syndrome.

**Educational value:**

Moving Ear Syndrome is effectively and safely treatable with EMG-US-guided botulinum toxin injections.

A 55-year-old man presented with brief backwards contractions of his right ear that began 4 months prior and progressively worsened, recurring several times per day. Ear movements were involuntary, partially suppressible, persistent during sleep, and associated with a feeling of “inner tension” and discomfort that radiated to the ipsilateral occipito-mastoid region (Video).

**Video:** Backwards sudden and brief right ear movements superimposed on a spontaneous tonic contraction of the posterior auricularis muscle.

Medical history revealed cranio-facial trauma with brain injury at 11 years, which resulted in left parieto-temporal encephalomalacia and ipsilateral cranioplasty. Since the age of 53, the patient has been effectively treated with carbamazepine 800 mg/day for focal-onset seizures with right upper limb paresthesia.

Baseline needle electromyography revealed spontaneous tonic activity in the right posterior auricularis muscle with 500 ms bursts of normal motor unit potentials (longer than previously reported) observed during ear movements [1].

A diagnosis of Moving Ear Syndrome (MES) was formulated and ten units of onabotulimtoxin A were injected into the muscle using a combined electromyographic-ultrasound guidance. After two weeks, discomfort resolved, and both frequency and amplitude of ear movements decreased, disappearing during sleep and occurring rarely during the day. At 12 weeks, while ear movements were slowly resuming during the day, a follow-up electromyography displayed a significant reduction in spontaneous tonic activity with occasional bursts(Supplementary figure).

The external ear muscles (anterior, superior, posterior) are vestigial muscles innervated by the facial nerve, which only 1 in 5 people can voluntarily activate. MES is a rare hyperkinetic disorder characterized by involuntary contractions of the external ear muscles, that may be rhythmic (1–2 Hz), unilateral or bilateral, and associated with pain [1]. Some authors have labeled ear movements as tics for partial voluntary suppressibility [1], while others described them as dystonic or myoclonic phenomena [2]. MES has been associated with neuroactive drugs including paroxetine, bromopride, chlorpromazine, and methylphenidate [2].

In this case, the feeling of inner tension and partial voluntary suppression are consistent with tics, whereas the spontaneous tonic activation, persistence during sleep, and rapid improvement following botulinum toxin argue in favor of facial nerve hyperexcitability, which might be post-traumatic, yet without neurogenic findings at needle electromyography.

Given the complex pathophysiology of MES, traditional pharmacological treatments (clonazepam, baclofen, pregabalin, propranolol) may be unsatisfying, while botulinum toxin and the anecdotal use of pallidothalamic tractotomy have proven to be effective [3].

Notably, the use of ultrasound combined with electromyographic guidance for botulinum toxin injections can enhance the precision of administration and reduce the risk of vascular injury. Although MES is rare and not typically disabling, early treatment can significantly improve quality of life with minimal risks.

## Data Accessibility Statement

The data that support the findings of this study are not openly available due to reasons of sensitivity and are available from the corresponding author upon reasonable request.

## Additional File

The additional file for this article can be found as follows:

10.5334/tohm.951.s1 Supplementary Figure. **Electromyography traces and ultrasound images.** Concentric needle electrode electromyography of right auricularis posterior muscle with top trace (1s, 200 μV) displaying abnormal tonic activity before botulinum toxin treatment and bottom trace (2s, 200 μV) displaying a significant reduction of tonic activity at 12-weeks follow-up with the persistence of occasional bursts [A]; educational ultrasound image displaying skin, posterior auricular muscle (PAM), and temporal bone (TB) [B].
