# Supplementary figures and images for: Echography-guided Botulinum Toxin for Moving Ear Syndrome
Source: Tremor Other Hyperkinet Mov (N Y). 2024 Nov 11;14:56. doi: 10.5334/tohm.951 (PMC11568798; doi:10.5334/tohm.951)

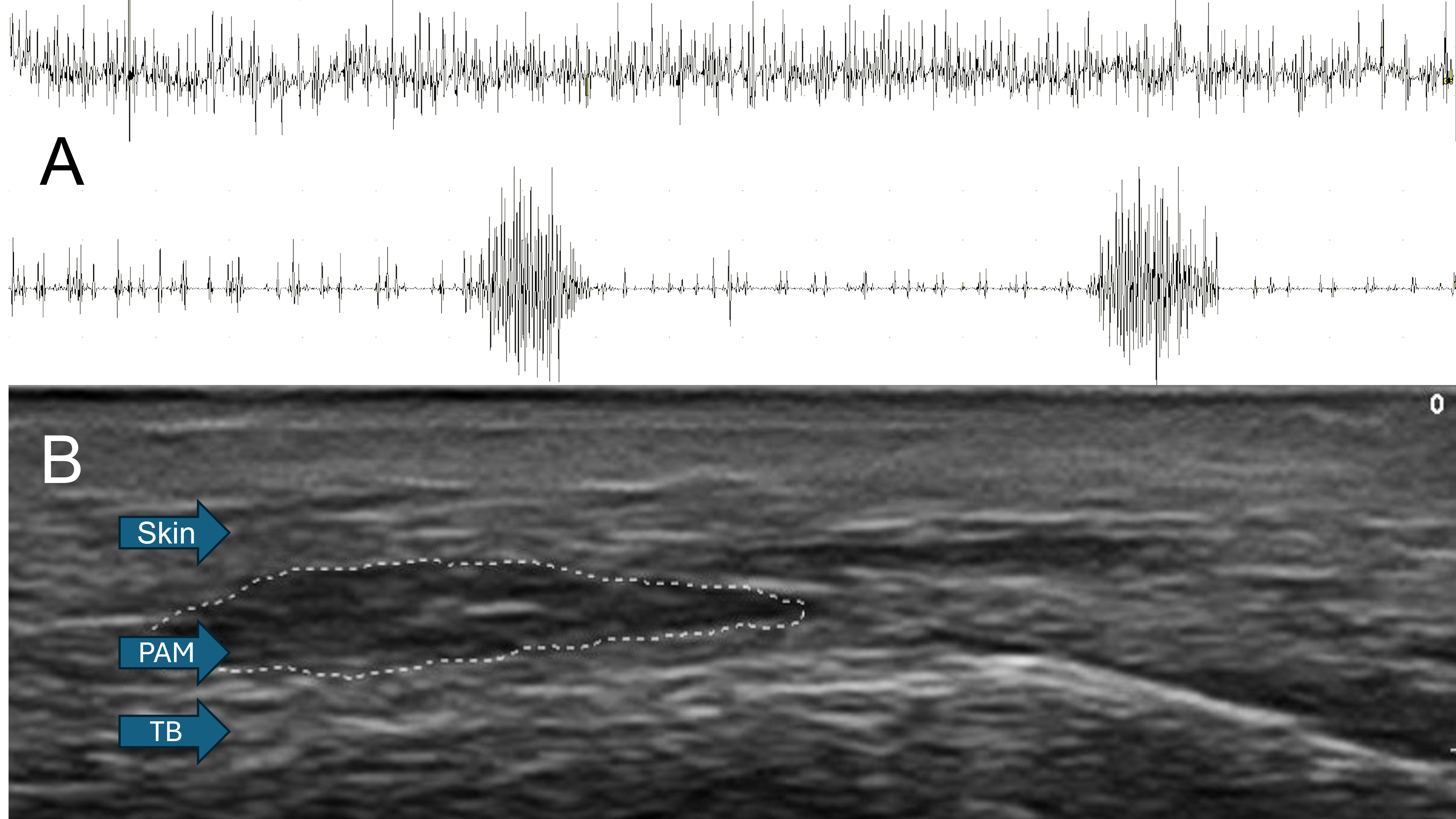

Supplement: Supplementary Figure. — Electromyography traces and ultrasound images. Concentric needle electrode electromyography of right auricularis posterior muscle with top trace (1s, 200 μV) displaying abnormal tonic activity before botulinum toxin treatment and bottom trace (2s, 200 μV) displaying a significant reduction of tonic activity at 12-weeks follow-up with the persistence of occasional bursts [A]; educational ultrasound image displaying skin, posterior auricular muscle (PAM), and temporal bone (TB) [B]. [file tohm-14-1-951-s1.tiff]
